# 21614 Use of an Outcomes Continuum to Describe Disparities in CVOID-19: Data from the OneFlorida Research Consortium

**DOI:** 10.1017/cts.2021.474

**Published:** 2021-03-30

**Authors:** Osama Dasa, Scott Cohen, Ruba Sajdeya, Yi Zheng, Mohamad B. Taha, Thomas A. Pearson

**Affiliations:** 1 University of Florida College of Medicine and Public Health and Health Professions; 2 University of Florida College of Public Health and Health Professions

## Abstract

ABSTRACT IMPACT: Identifying differential COVID-19 progression across the disease continuum may help policymakers and service providers better identify or predict gaps in services and resources and develop precision strategies to support COVID-19 patients where the need is mostly needed. OBJECTIVES/GOALS: Single institution studies have documented COVID-19 disproportionally affected US racial and ethnic minority groups compared to Whites. However, few population-wide data studied severity and death in multiracial populations. We aim to examine the current disparity in the COVID-19 continuum, including hospitalizations, severity, and death. METHODS/STUDY POPULATION: Data on 67,094 laboratory documented COVID-19 cases nested from the state-wide ‘OneFlorida’ research consortium through August 3, 2020, were assessed to decide differences and disparities in COVID-19 outcomes. A COVID-19 outcome continuum outlining the proportions of cases transitioning from diagnosis to death was constructed (Figure 1). OneFlorida partners provide health care to more than 40% of Floridians in the nation’s third-largest and very diverse state. OneFlorida partners encompass hospitals, practice/clinic settings, and physicians, which provide care for 15 million patients across all of Florida’s 67 counties. It is part of the Patient-Centered Outcomes Research Institute (PCORI). RESULTS/ANTICIPATED RESULTS: Among cases, 25,443 (37.9%) were non-Hispanic Whites, 11,709 (17.5%) were non-Hispanic Blacks, and 16,119 (24.0%) were Hispanics. Among COVID-19 patients, Blacks and Hispanics had a higher frequency of emergency department (ED) visits (45.7% and 46.0%, respectively), whereas admission rates were higher in Blacks (15.6%) and Whites (15.9%) than in Hispanics (11.5%). Blacks had the highest rates of intubation (3.6%) and in-hospital deaths (2.7%) compared to Whites (2.5% and 2.3%, respectively) and Hispanics (1.3% and 1.4%, respectively), Figure 1. When rates were indexed to the state census data, Blacks had the worst rates across the disease continuum (infection to death). In comparison, Hispanics had higher rates of ED visits but lower rates of intubation and death, Table 1. DISCUSSION/SIGNIFICANCE OF FINDINGS: Outcomes continuum is a useful tool at an individual-level to assess care outcomes and at population-level as a framework to analyze the proportion of population with COVID-19 that progress to each successive disease stage. This will help policymakers to better identify gaps in services and develop precision strategies to support COVID-19 patients.

